# Role of Oxidative Stress in Refractory Epilepsy: Evidence in Patients and Experimental Models

**DOI:** 10.3390/ijms14011455

**Published:** 2013-01-14

**Authors:** Noemi Cardenas-Rodriguez, Bernardino Huerta-Gertrudis, Liliana Rivera-Espinosa, Hortencia Montesinos-Correa, Cindy Bandala, Liliana Carmona-Aparicio, Elvia Coballase-Urrutia

**Affiliations:** 1 Laboratory of Neurochemistry, National Institute of Pediatrics, 04530, D.F., Mexico; E-Mails: noemicr2001@yahoo.com.mx (N.C.-R.); osoger2065@yahoo.com.mx (B.H.-G.); 2 Laboratory of Pharmacology, National Institute of Pediatrics, 04530, D.F., Mexico; E-Mail: lili_rives@yahoo.com; 3 Department of Endocrinology, National Institute of Pediatrics, 04530, D.F., Mexico; E-Mail: hortenciamontesinoscorrea@yahoo.com; 4 Unit of Support to Research, National Institute of Rehabilitation, 14389, D.F., Mexico; E-Mail: cindimiel@hotmail.com

**Keywords:** oxidative stress, free radicals, refractory epilepsy, pharmacoresistant epilepsy

## Abstract

Oxidative stress, a state of imbalance in the production of reactive oxygen species and nitrogen, is induced by a wide variety of factors. This biochemical state is associated with systemic diseases, and diseases affecting the central nervous system. Epilepsy is a chronic neurological disorder with refractoriness to drug therapy at about 30%. Currently, experimental evidence supports the involvement of oxidative stress in seizures, in the process of their generation, and in the mechanisms associated with refractoriness to drug therapy. Hence, the aim of this review is to present information in order to facilitate the handling of this evidence and determine the therapeutic impact of the biochemical status for this pathology.

## 1. Introduction

Oxidative stress is a biochemical state in which reactive oxygen species are generated. Since the 1970s, it has been associated with diverse physiological and pathological states, including diseases like epilepsy that affect the central nervous system.

Epilepsy is a chronic neurological disease that affects up to 1% of the world population, with greater incidence at early ages. Approximately 30% of patients suffering from this condition present resistance to pharmacological treatment. Since the 1990s, ever-greater attention has been paid to biochemical alterations, and specifically to the role played by the oxidative state, in the effort to identify the mechanisms underlying the pathological development of refractory epilepsy.

## 2. Reactive Oxygen Species (ROS) and Reactive Nitrogen Species (RNS); Oxidative Stress and Antioxidant Defense Mechanisms: Biological Importance

It is known that reactive oxygen species (ROS) and reactive nitrogen species (RNS) can be both beneficial and harmful in biological systems. The beneficial effects of ROS are evident in its physiological role in numerous cellular responses and cell signaling systems. In contrast, at high concentrations, ROS may be important mediators of damage to various cellular structures: lipids, proteins, and nucleic acids. The beneficial effects of ROS are supplemented by the action of non-enzymatic antioxidants, as well as the antioxidant enzyme system. Despite the presence of the antioxidant defense system to combat oxidative damage caused by ROS, such damage is accumulated during the entire life cycle of organisms. Imbalance between the production of ROS/RNS and antioxidant defense mechanisms or oxidative stress plays a very important role in the development of many diseases, such as cancer, atherosclerosis, arthritis, neurodegenerative disorders, and other conditions [1].

### 2.1. Oxidative Stress

#### 2.1.1. Free Radicals

Free radicals (FR) can be defined as molecules or molecular fragments, capable of independent existence, and containing one or more unpaired electrons [1,2]. The presence of unpaired electrons in FR usually confers to them a considerable degree of reactivity to FR. They can be generated by: (a) the loss of an electron to a non-radical; (b) the acquisition of an electron from a non-radical; or (c) the homolytic cleavage of a molecule.

The presence of oxygen-derived FR represents the most important source of these species generated in living systems [3]. The term “reactive oxygen species” is a collective term that involves oxygen-derived FR, as well as non-radicals derived from the reduction of molecular oxygen (O_2_) (see Table 1).

The sources of ROS include mitochondria, cytochrome P450 system, peroxisomes and activation of inflammatory cells [4]. The mitochondria is one of the sites which generates significant amounts of H_2_O_2_.

#### 2.1.2. Formation of Some ROS

Superoxide (O_2_^•−^) is formed by the univalent reduction of oxygen. Despite being relatively unstable, it can be held in equilibrium by its conjugate acid, the hydroperoxyl radical. O_2_^•−^ has an important function *in vivo*, and participates in the respiratory burst of activated phagocytes in immunological events. When these cells are activated, the NADPH oxidase enzyme complex, located in the cytoplasmic membrane, partially reduces O_2_ according to the following reaction [2,5]:

$$2{\text{O}}_{2}+\text{NADPH}+{\text{H}}^{+}\to {{\text{O}}_{2}}^{•-}+{\text{NADP}}^{+}+2{\text{H}}^{+}$$

Similarly, xanthine oxidase (XO) is capable of reducing O_2_ to O_2_^•−^ as follows:

$$\text{Xanthine}+{\text{O}}_{2}+{\text{H}}_{2}\text{O}\to \text{Uric acid}+{{\text{O}}_{2}}^{•-}+{\text{H}}^{+}$$

Hydrogen peroxide (H_2_O_2_), formed by the superoxide dismutase (SOD) enzyme, has great lipophilicity and crosses cell membranes to react with O_2_^•−^ in the presence of transition metals to generating HO·. For this reason, it is an important oxidant in aerobic organisms [6].

$$2{{\text{O}}_{2}}^{•-}+2{\text{H}}^{+}\to {\text{O}}_{2}+{\text{H}}_{2}{\text{O}}_{2}$$

The hydroxyl radical (HO·) is considered one of the most harmful oxidative species due to its short half-life and high reactivity. HO· is generated by the Fenton and Haber–Weiss reaction between O_2_^•−^ and H_2_O_2_, catalyzed by a transition metal [2,6].

$$\begin{array}{c} {\text{Fe}}^{2+}/{\text{Fe}}^{3+}:\\ {{\text{O}}_{2}}^{•-}+{\text{H}}_{2}{\text{O}}_{2}\to {\text{O}}_{2}+{\text{OH}}^{-}+\text{HO}\cdot \end{array}$$

HO may also be formed from HOCl,

$$\text{HOCl}+{{\text{O}}_{2}}^{•-}\to \text{HO}\cdot +{\text{Cl}}^{-}+\Delta^{1}{\text{O}}_{2}$$

The peroxynitrite anion (ONOO^−^) can be formed by the combination of O_2_^•−^ and nitric oxide (NO·) [7]: NO· + O_2_^•−^ → ONOO^−^. It is a powerful oxidant which induces tyrosine nitration, lipid peroxidation and cytotoxicity, and is involved in several pathological conditions [8,9].

Hypochlorous acid (HOCl) is produced by the reaction of peroxidases with H_2_O_2_. For instance, myeloperoxidase (MPO), present in high concentrations in the granules of neutrophils, catalyzes the conversion of H_2_O_2_ to HOCl according to the following reaction [10]:

$${\text{Cl}}^{-}+{\text{H}}_{2}{\text{O}}_{2}\to \text{HOCl}+\text{HO}\cdot$$

Ozone, a pale blue gas abundant in the upper layers of the atmosphere, is not a FR. It is produced by the photodissociation of O_2_ into two oxygen atoms, each of which may react with a molecule of O^2^ [2,11]:

$$\begin{array}{c} E\\ {\text{O}}_{2}\to 2\text{O}\\ {\text{O}}_{2}+\text{O}\to {\text{O}}_{3} \end{array}$$

#### 2.1.3. Cellular and Extracellular Antioxidant Defense Mechanisms

An antioxidant is any substance that at relatively low concentrations, in relation to the oxidizable substrate, significantly delays or prevents oxidation of the substrate [2]. The physiological production of ROS in aerobic organisms requires the presence of a defense system against effects of these oxidative species. This defense system includes two parts: high molecular weight and low molecular weight antioxidants.

##### 2.1.3.1. High Molecular Weight Antioxidants

Superoxide dismutase (SOD): Cu-Zn SOD and Mn-SOD: This enzyme is found in the cytoplasm (Cu-Zn SOD), which is a homodimer of 32.5 kDa, containing Cu (II) and Zn (II) in the active site to form a bridge with imidazole or histidine. Mn-SOD is located in the mitochondrial matrix and is a 95 kDa homotetramer containing Mn (II) in its active site. [1,12,13].

SOD, as shown above, catalyzes the conversion of O_2_^•−^ to H_2_O_2_.

Catalase (CAT): This enzyme, a tetramer of about 240 kDa, is found mainly in mitochondria and peroxisomes, and catalyzes the dismutation of hydrogen peroxide to water and oxygen [14]:

$$2{\text{H}}_{2}{\text{O}}_{2}\to 2{\text{H}}_{2}\text{O}+{\text{O}}_{2}$$

Glutathione peroxidase (GPx): This enzyme catalyzes the reduction of two molecules of peroxide using reduced glutathione (GSH). The products of the reaction are oxidized glutathione (GSSG) and water [15].

$$\begin{array}{c} {\text{H}}_{2}{\text{O}}_{2}+2\text{GSH}\to \text{GSSG}+{\text{H}}_{2}\text{O}\\ \text{ROOH}+2\text{GSH}\to \text{GSSG}+\text{ROH}+{\text{H}}_{2}\text{O} \end{array}$$

This selenoenzyme is found in several isoforms [16–19]. Cytosolic GPx (cGPx, GPx-1) is present in the cytosol of all tissues, although at different distribution. Plasma GPx (pGPx, GPx-3) exists in the extracellular fluids of various tissues, and in high concentration in the kidney. Phospholipid GPx (PHGPx, GPx-4) in the membrane and cytosol of various tissues, functions as an antioxidant in the cell membrane. It is abundant in testicles. Gastrointestinal GPx (GI-GPx, GPx-2) is present in the cytosol of the liver and the intestinal tract in humans.

Glutathione reductase (GR): This enzyme is found in the cytoplasm and has a FAD^+^ coenzyme in its active site. It catalyzes the reduction of GSSG using coenzyme NADPH [20]:

$$\text{GSSG}+\text{NADPH}+{\text{H}}^{+}\to {\text{NADP}}^{+}+2\text{GSH}$$

Evidence indicates that the NADPH reduces FAD^+^, which transfers two electrons to the disulfide linkage (–S–S–) between two cysteine residues of the active site. The two-SH groups then formed interact with GSSG, reducing to two molecules of GSH. GR maintains GSH levels in the cell [21].

Glutathione-S-transferase (GST): Cytosolic GST is divided into four main families (α, μ, π and θ), four minor families (δ, σ, κ and ω). Cytosolic GST consists of two identical protein subunits, whereas microsomal GST is a trimmer [22–24].

The principal function of this enzyme is the conjugation of glutathione with many organic compounds, according to the following reaction:

RX+GSH→RSG+HX

GST can reduce lipid hydroperoxides (such as GPx) and may also detoxify 4-hydroxynonenal (4-HNE), a product of lipid peroxidation.

Thioredoxin (Trx): This enzyme, with a molecular weight of 12 kDa, is mainly found in the endoplasmic reticulum [25]. It contains two adjacent thiol groups in its reduced form (SH), which can be oxidized to form disulfide (S_2_ or S–S):

$$\text{Trx}-{\left(\text{SH}\right)}_{2}+{\text{protein}-\text{S}}_{2}\to {\text{Trx}-\text{S}}_{2}+\text{protein}-{\left(\text{SH}\right)}_{2}$$

Trx can provide electrons in various redox reactions and, in the same manner, can react directly with H_2_O_2_:

$$\text{Trx}-{\left(\text{SH}\right)}_{2}+2{\text{H}}_{2}{\text{O}}_{2}\to {\text{Trx}-\text{S}}_{2}+2{\text{H}}_{2}\text{O}+{\text{O}}_{2}$$

Thioredoxin reductase catalyzes the reduction of the Trx polypeptide [26]:

$${\text{Trx}-\text{S}}_{2}+\text{NADPH}+{\text{H}}^{+}\to {\text{NADP}}^{+}+\text{Trx}-{\left(\text{SH}\right)}_{2}$$

Peroxiredoxins (Prxs): This group of enzymes, dimeric proteins of approximately 23 kDa, are characterized by cysteine residues in their catalytic center, and act as antioxidants specific to thiol groups. These proteins are also involved in the enzymatic degradation of H_2_O_2_, hydroperoxides and ONOO^−^ [27,28].

$$\text{Prx}{\left(-\text{SH}\right)}_{2}+2{\text{H}}_{2}{\text{O}}_{2}\to {\text{Prx}-\text{S}}_{2}+2{\text{H}}_{2}\text{O}+{\text{O}}_{2}$$

Trx can regenerate the reduced form of the Prx:

$${\text{Prx}-\text{S}}_{2}+\text{Trx}-{\left(\text{SH}\right)}_{2}\to \text{Prx}{\left(-\text{SH}\right)}_{2}+{\text{Trx}-\text{S}}_{2}$$

##### 2.1.3.2. Low Molecular Weight Antioxidants

Low molecular weight antioxidants are generally classified as hydrophilic (especially vitamin C, GSH and uric acid) and lipophilic (especially vitamins A and E and bilirubin). Both vitamin C and vitamin E are obtained from the diet. Other known antioxidants that are also obtained from some foods (particularly fruits and vegetables) include carotenoids (*i.e.*, lycopene, lutein and beta-carotene) and phenolic compounds (*i.e.*, flavonols, flavones, flavanones, anthocyanidins and phenylpropanoids) [29,30].

Experimental evidence suggests that the capacity of flavonoids to act as an antioxidant is dependent upon their molecular structure, the position of hydroxyl groups and other substitutions in the chemical structure of these polyphenols, likewise these positive effects have been attributed to free radical scavenging, transition metal chelation, activation of survival genes and signaling pathways, regulation of mitochondrial function, and modulation of neuroinflammation [31–38].

### 2.2. Participation of Oxidative Stress in Diseases of the Central Nervous System

All aerobic organisms are susceptible to oxidative stress, since oxygen species such as O_2_^•−^ and H_2_O_2_ are produced in the mitochondria [39]. The brain is considered highly sensitive to oxidative damage [40], which can be explained by the fact that it contains a large amount of readily oxidizable fatty acids (20:4 and 22:6) and has poor antioxidant defenses [41].

The first experimental evidence that related oxidative stress with diseases of the central nervous system (CNS) was described by Kosiakov *et al*. [42] who found differences between the level of hemoglobin and the CAT index in human brain tumors and in healthy individuals [42]. At present, there is evidence to show that the production of FRs is associated with damage to cell structures during the pathogenesis of the following diseases of the CNS:

- In patients with Parkinson’s disease (PD), FRs have been associated with several genes, such as SNCA, parkin, DJ-1, PINK1 and LRRK2, with a defect in complex I of the mitochondrial electron-transport chain and oxidative stress in substantia nigra [39,43]. Moreover, in experimental models, an excessive formation of ROS was found to lead to increased lipid peroxidation, oxidative damage of DNA, GSH depletion and enhanced SOD activity [43–50].
- In patients with Alzheimer’s disease, lipid peroxidation products and increased Aβ production have been shown to induce c-Jun-*N*-terminal-Kinase (JNK) pathways, leading to neuronal apoptosis, as well as to the production of 4-HNE, malondialdehyde (MDA), and the α,β-unsaturated aldehyde (acrolein). The latter are diffusible and highly reactive with other biomolecules, and are consequently neurotoxic [51–54].
- In patients with amyotrophic lateral sclerosis, several mechanisms have been implicated, including glutamate excitotoxicity; aberrant protein aggregates containing mutant Cu-Zn-SOD, mislocalization and aggregation of neurofilaments; oxidative damage by enhanced FR formation; and, mitochondrial dysfunction [55–60]. Recent evidence highlights the Peroxisome Proliferator-Activated Receptors (PPARs) as critical neuroprotective factors in ALS. PPARs can be activated by lipid peroxidation metabolites in ALS motor neurons, and this can prompt the expression of antioxidant enzymes such as GPx, GR, and Cu-Zn-SOD [61,62].
- In epileptic patients, there are several mechanisms related to FR production, which will be described in the following section.

## 3. Role of Oxidative Stress in Refractory Epilepsy

### 3.1. Epilepsy in General

The word “epilepsy” derives from the Greek verb *epilamvanein*, meaning “to be overwhelmed suddenly” or “to be taken by surprise” [63]. Epidemiological studies indicate that this pathology affects 0.5 to 1% of the world population [64–67]. The incidence is higher in childhood, stabilizes in adulthood, and increases in the last decades of life [66,68]. Epilepsy is a disorder of the CNS characterized by increased and abnormal synchronization of neuronal electrical activity, which is manifested by recurrent seizures, as well as spontaneous and unpredictable excessive seizures known as a stroke [63]. This abnormal electrical activity in epilepsy may be subclinical and therefore detectable by electroenchephalography (EEG) [69–71]. Thus, this technique represents a basic method of differential diagnosis [71].

#### 3.1.1. Classification of Seizures

The term “seizure” refers to a behavioral change (impaired motor and/or sensory activity) because of synchronicitis, which is an alteration in the rhythmic firing of a population of neurons [63,72,73].

Seizures are classified based on their etiology: (a) idiopathic or primary, which are associated with hereditary predisposition; (b) secondary or symptomatic, associated with any event that damages the brain, such as head trauma, benign or malignant tumors, bleeding, infections (meningitis, encephalitis or abscesses), vascular malformations, and metabolic causes; and (c) cryptogenic, whose origin is unknown [73–76]. Based on the location of the hypersynchronous activity of neurons, seizures are classified as partial or generalized [75–78]. Partial seizures are the result of an abnormal electrical discharge in a specific part of the brain (seizure focus), and therefore are also known as focal seizures. These can be simple (no change in consciousness), complex (with decrease or loss of consciousness) and partial. They become widespread when abnormal electric activity spreads to the entire brain [70]. Complex partial seizures originate preferentially in temporal lobe structures (hippocampus, amygdala, temporal neocortex, *etc.*) [72,75–78].

Generalized seizures are those in which there is an increase of abnormal electrical activity occurs in both cerebral hemispheres simultaneously [68,72]. Such seizures can lead to generalized motor impairment, which may be accompanied by autonomic discharge. The latter is evidenced by an EEG pattern that is bilateral, synchronous and symmetrical in both hemispheres [76,78].

#### 3.1.2. Refractory Epilepsy

There is currently no general consensus on the definition of refractory epilepsy, which is very difficult to control. In the literature, many terms are used synonymously (drug-resistant epilepsy, intractable epilepsy, refractory epilepsy, as well as severe and disabling epilepsy) [79–81]. Most authors agree that this condition is one that does not respond to the use of two or three first-line antiepileptic drugs at the maximum tolerable dose (either alone or in combination), over a period of one to two years. Others define it as the persistence of one or more seizures per month in spite of a treatment with at least two drugs [82–84].

##### Mechanisms Associated with Refractory Epilepsy

Drug resistance in patients with epilepsy may be due to mechanisms of pharmacokinetic or pharmacodynamic origin:

1. Pharmacokinetic mechanisms (absorption, metabolism and elimination) are those which enable the attainment of antiepileptic drug concentrations at the site of action. They include inadequate dose administration (pseudo-resistance), subtherapeutic serum concentration, despite an appropriate dose, and the insufficient concentration of the active substance in the brain parenchyma, even when serum levels are within the therapeutic range. The biological effect of an antiepileptic drug is determined by the physical properties of the active principle. One of the most important properties is lipid solubility, which affects distribution in the different compartments of the CNS. In general, pharmacokinetic mechanisms influence absorption, metabolism and elimination of the drug with its metabolites [82,83,85–90].
2. Pharmacodynamic mechanisms resistance alters the action of antiepileptic drugs at their sites of action (target sites) in the CNS despite adequate serum levels and concentrations in cerebral parenchyma. Pharmacodynamic alterations may occur at the synapses (site of neuronal communication) or at the effector sites located in the neuronal membrane (ion channels and receptors) [82,83].

In general, we suggest three hypotheses to try to explain pharmacodynamic alterations:

1. The carrier hypothesis emphasizes the seizure focus, where there is an increased expression of proteins that can carry pharmaceuticals. These proteins are mainly P-glycoproteins (PgP) and their increased expression is due to changes in the acquired epileptic focus or because of a genetic variation the gene that encodes it. The drug-refractory state is related to an increased expression of a gene involved in multidrug resistance (MDR, “Multidrug Resistence”) and the gene encoding PgP. The latter is an ATP-dependent protein, which exports drugs and toxic material from the cells into the bloodstream. That is, there is no transport of the drug out of the epileptic focus. These proteins, predominantly expressed in both endothelial cells and astrocytes, regulate the capacity of antiepileptic drugs to cross the blood–brain barrier and the blood-cerebral spinal fluid (CSF) barrier. As a result of the upregulation of PgP, decreased concentrations of phenytoin and oxcarbazepine have been observed in epileptic foci. It is possible that the intracerebral administration of verapamil and PSC833 (PgP inhibitors) could avoid the extracellular transport to the bloodstream of antiepileptic drugs, which would thus facilitate their action in the brain [89,91–96].
2. The modification of the drug targets hypothesis on changes in receptor expression, or the amount of ion channels at the cellular level within the epileptic area, makes it less susceptible to antiepileptic drugs. For example, changes have been observed in sodium channels and voltage-dependent calcium channels, as well as Gamma-aminobutyric acid-A (GABA-A) receptors, leading to reduced efficacy of various antiepileptic drugs. These alterations in the structure and/or functionality of the drug target of antiepileptic drugs in the brain can be intrinsic (genetic) and acquired (disease-related) [82,88,92,95].
3. The hypothesis called the intrinsic gravity model of epilepsy proposes that there is a continuum of disease severity that determines the patient’s relative response to medication. According to recent studies, a high frequency of seizures in the early phase of epilepsy is the dominant risk factor influencing the possibility of remission of seizures. This risk factor surpasses the contribution of other possible factors associated with prognosis, including the etiology of epilepsy, seizure type, or the result of EEG or imaging. Thus the failure of antiepileptic drugs can be explained, according to this hypothesis, by evaluating differences in the severity of epilepsy based on the frequency of seizures during the early stages of the disease [88,97].

The first two hypotheses have been criticized, and have their obvious weaknesses. The carrier hypothesis implies that all antiepileptic drugs act on transport proteins, because patients with refractory epilepsy are resistant to all drugs. However, there is evidence that all antiepileptic drugs are substrates of PgP. Moreover, human P-glycoprotein may not present the same activity as PgP evaluated in animal models. Likewise, the modification of the drug targets hypothesis is undermined by the fact that patients are resistant to drugs that have different mechanisms of action, and therefore would be affected differently by any of these mechanisms. Drug resistance is a complex problem and one theory is probably not sufficient to explain the phenomenon. Maybe epilepsy deteriorates over time, implying that severity is due to the progression of the disease [97].

Finally, other mechanisms associated with refractory epilepsy, such as oxidative stress, should not be excluded from consideration. It is still not known if oxidative stress is a cause or consequence of this pathology.

### 3.2. Evidence of Participation of Oxidative Stress in Refractory Epilepsy

Oxidative stress was first associated with neuronal hyperexcitation caused by diseases of the nervous system in the early 1990s [98,99]. Dalton *et al*. [100] was the first to determine that, in a rat model of epilepsy (treating the brain with kainic acid), severe brain damage was in part caused by oxidative stress. They found that kainic acid induced an increase in metallothionein-I and heme oxygenase-I mRNA, as well as an increase in c-fos, heat shock protein-70, and interleukin-1 beta mRNA, with little or no change in the mRNA for metallothionein-III, Mn-SOD, Cu-Zn-SOD, GST and GPx. The induction of the metallothionein-I and heme oxygenase-I gene expression suggests that oxidative stress is produced by kainic acid-induced seizures. Furthermore, the induction of interleukin-1 beta gene expression suggests that there is an inflammatory response in brain regions damaged by kainic acid-induced seizures [100]. The results of this study have encouraged further research into the possible role of oxidative stress in epilepsy and refractory epilepsy.

#### 3.2.1. Evidence from Experimental Models

No direct data exists in regards to the possible participation of oxidative stress in refractory epilepsy in patients or animal models. However, indirect evidence has been found from two types of studies: (i) the attempt to determine the biological mechanism that underlie the ketogenic diet (an alternative therapy for refractory epilepsy); and (ii) the analysis of the effect of Cystatin B in regulating redox in a model of progressive myoclonus epilepsy (a form of epilepsy with a high frequency of refractoriness).

1. The ketogenic diet has been used for almost 80 years in the treatment of refractory epilepsy, although the biochemical mechanisms involved are unknown. Ziegler *et al*. [101] studied the role of the oxidative stress in the effect of this kind of diet on Wistar rats, determining lipoperoxidation levels and enzyme activities of GPx, CAT, and SOD in different brain regions of Wistar rats fed a ketogenic diet. They observed that there were no changes in cerebral cortex, but that in the cerebellum, there was a decrease in total antioxidant capacity, measured by a luminol oxidation assay, in spite of the lack of change in antioxidant enzyme activity. In the hippocampus, they observed an increase in antioxidant activity with an approximately four-fold increase of GPx accompanied by no change in lipoperoxidation levels. These results were the first to suggest that the higher activity of this enzyme in the hippocampus induced by the ketogenic diet could be a mechanism of protection against neurodegenerative damage normally induced by convulsive disorders in this structure [101].
2. Lehtinen *et al.* [102] studied the participation of Cystatin B, an inhibitor of lysosomal Cathepsins, primary genetic cause of the Unverricht-Lundborg type (EPM1), part of progressive myoclonus epilepsies (PME). They studied the role of Cystatin B (an inhibitor of lysosomal Cathepsins) in regulating redox homeostasis and oxidative stress responses in transfected mice, using a plasmid-based method of RNA. Rat granule neurons were transfected with the Cystatin B hpRNA or control U6 plasmid, together with the β-galactosidase expression plasmid. First, they exposed transfected neurons to oxidative conditions before inoculating them into mice. In the neurons transfected with Cystatin B, the damage induced by exposure to oxidants was reduced, evidenced by a decrease in the percentage of cell death. After this experiment, the protective capacity of transfecting neurons with Cystatin B was tested in knockout mice, observing the effects on the Cystatin B −/− gene in oxidative conditions. It was found that these transfected neurons were protected from oxidative damage that can produce cell death (in the presence of H_2_O_2_ and glutamate).

This research group proposed that the deficiency of Cystatin B in cerebellum made the animals more susceptible to oxidative damage. Since this could be a factor contributing to the pathogenesis of EPM1, the levels of some antioxidant enzymes (SOD, CAT and GSH) associated with the process of neurodegeneration were determined. Additionally, the level of lypoperoxidation was determined. The animals presented an increase in lypoperoxidation, a decrease in the levels of SOD and GSH, and no change in CAT. These results suggest that a deficiency in Cystatin B increases susceptibility of cerebellum to oxidative damage, and especially to lypoperoxidation. Additionally, it was shown that Cystatin B is a gene inducible by oxidative conditions, through the activation of the transcription factor Sp1 (a factor associated with the inducible transcriptional response to oxidative stress), which in turn binds to a promoter of Cystatin B.

Finally, the activity of Cystatin B was determined in culture mediums of granular neurons from cerebellum deficient in Cystatin B, finding an increase in this inhibitor. It was also shown that neurodegeneration of granular neurons was greater in double knockout mice (Cystatin B and Cathepsin B) than knockout mice (Cystatin B). These results suggest that there is an increase in the probability that Cystatin B can protect neurons against damage by oxidative stress if Cathepsin B is inhibited, and therefore evidence the participation of Cystatin B in the mechanisms involved in the pathogenesis of EPM1. Hence, Cystatin B could likely be an effective therapeutic target for treating this pathology [102].

#### 3.2.2. Studies in Patients

Clinical studies to determine the participation of oxidative stress in epilepsy have focused on patients with progressive myoclonus epilepsies [103], refractory status epilepsy in the presence of encephalopathy [104], and refractory epilepsy [105].

(1) Ben-Menachem *et al.* [103] determined the levels of different antioxidant enzymes in the blood of patients with progressive myoclonic epilepsies (EPM; a type of epilepsy with difficult therapeutic management and refractoriness to most antiepileptic drugs) and compared these levels with those in healthy controls. The plasma of these patients was analyzed, to determine the levels and activity of GPx, CAT and Cu–Zn–SOD, and compare these parameters with those of healthy controls. It was found that the activity of Cu–Zn–SOD in EPM patients was lower than in controls. Under the assumption that the decreased activity of this enzyme facilitates damage by oxidative stress and therefore the neurological pathology, pretreatment was performed with an antioxidant called *N*-acetylcysteine (NAC) at high doses (6 g/día) in patients with EPM. The results obtained in this study suggest that NAC was overall favorable in EPM patients. This was the first evidence that epilepsy is difficult to control, and that drug-resistant epilepsy could be treated with antioxidants to improve the condition of patients, thereby emphasizing the need to understand the role of oxidative stress in epilepsy refractory to drug treatment [103].

(2) Shiihara *et al.* [104] reported a case of a patient with acute encephalopathy with refractory status epilepticus, showing bilateral mesial temporal and claustral lesions that could be related to DNA oxidative damage. These authors studied a 12-year-old girl, who was treated with theophylline for bronchial asthma for about three years. This produced frequent tonic convulsions in the absence of a history of neurological disorders or any contributory family history. The patient was treated with conventional antiepileptics such as phenobarbital, phenytoin and valproate, as well as with anesthetic agents, including midazolam, thiamylal and propofol. However, all of these efforts failed to produce the desired effect. Therefore, the patient was administered thiopental, which was suspended on day 82 due to side effects, including leukopenia, hypotension, atelectasis, thrombophlebitis, renal failure and liver damage. Afterward, the patient was treated with phenitoin and high doses of phenobarbital to reduce the frequency and intensity of seizures.

The authors investigate the causes of the patient crisis attributed to the theophylline administration, such as the possible trigger of the crisis supported by experimental evidence where it described that theophylline induced seizures in mice and is therefore associated with FR-produced oxidative stress. Consequently, the authors determine to quantify the deoxyguanosine (stress oxidative biomarker). Samples were obtained of cerebral–spinal fluid, blood and urine to determine 8-OHdG after convulsions started. The results showed increased 8-OHdG in a sample of cerebral–spinal fluid on day 10, and in a sample of plasma and urine on day 23 after seizures started. In rat experimental models, systemic kainic acid, which induces seizures, increased cerebral 8-OHdG levels, up to seven-fold within 72 h. This was compatible with the patient.

Additionally, other studies like EEG, cranial computed tomography (CT) and magnetic resonance imaging (MRI) were used to determine the alterations in the brain. The EEG studies showed multifocal spikes or sharp waves, tending to burst. A cranial MRI on day 7 revealed no abnormalities, but on day 27, demonstrated bilateral mesial temporal and claustral lesions.

Oxidative DNA damage could be evoked within a few days after epilepticus status begins, and neuroimaging changes may continue for some time beyond this. The researchers speculate that augmented oxidative stress was associated with refractory epilepticus status in the patient, because 8-OHdG is increased in the brain, including the amygdale and hippocampus, in the seizure-induced rat, and is associated with increased DNA fragmentation that results in neuronal cell death. Hence, serial measurements of oxidative stress markers in acute encephalitis, encephalopathy or status epilepticus could clarify the relationships between acute brain damage and FR [104,105].

(3) Since there are only a few reports of oxidative stress markers in the plasma and other fluids after epilepsy surgery, López *et al.* [106] investigated the impact of the epilepsy surgery on markers of oxidative damage in the serum of drug-resistant epileptic patients. Nine patients (five males and four females; ages 37 ± 6 years) were studied, all diagnosed with temporal lobe epilepsy (TLE) refractory to drugs. They were being administered the two major antiepileptic drugs, in two monotherapy cycles and at least one polytherapy. Patients were kept under their habitual drug treatment (drugs and doses) during the entire time of the study. Serum samples from 32 healthy individuals without any drug treatment were used as controls. The age range and gender distribution of the control group was similar to the group of patients (18 males and 14 females; ages 37 ± 9 years). The samples were used to determine the activity of antioxidant enzymes SOD, CAT and GPx, as well as the concentration of products of oxidative damage to lipids and proteins, MDA, and advanced oxidation protein products (AOPP), respectively.

It was found that, compared to control subjects, the epileptic pre-surgery phase presented altered antioxidant enzyme activity and increased levels of the oxidative damage marker. After surgery, the patients showed a tendency to normalization of the studied variables, except for SOD activity. The outlying redox state of the patients with TLE refractory to drugs markedly improved after surgery, which is clearly evidenced by an important decrease in MDA and AOPP levels two years after surgery. The recovery in GPx activity was also notorious, as it contributes to a decrease in oxidative damage and a better redox balance. However, one can speculate that the sustained increase in SOD activity could recede if the epileptoid activity in the remaining regions eventually disappeared in these patients. Finally, the increase in levels of CAT activity seems to be a cellular response to the intense ROS production triggered by seizure episodes [106].

## 4. Therapeutic Relevance

In recent years, the role of oxidative stress in diseases of the CNS has been attracting increasing attention. In such diseases, and particularly in refractory epilepsy, FR, mitochondrial DNA (mtDNA) mutation, decreased mitochondrial respiration, mitochondrial calcium dysregulation, lipid oxidation, protein modification, mitochondrial permeability transition, among other factors, are frequently observed, although to different degrees. In this review, we show that, in epilepsy, an imbalance exists in the antioxidant defense system. The decrease in oxidative stress using antioxidants could be a therapeutic option leading to the reduced administration and adverse effects of some anticonvulsants agents.

Experiments conducted in animals and cellular models, as well as observations of patients, all suggest the benefit of protection against oxidative damage for attenuation of neuronal degeneration [107–111]. Thus, it has been proposed that therapeutic approaches target oxidative stress in the treatment of diseases of CNS, including refractory epilepsy. For example, therapeutic benefits from antioxidant treatment with agents such as *N*-acetylcysteine (NAC) may be due to their capacity to stop or slow the process of neuronal cell death. There is a need for further studies in clinical trials with specific patient groups that have drug-resistant epilepsy. NAC should mainly function as a precursor of glutathione (a powerful antioxidant). This is important because it indicates that the NAC effect is more general and can improve the condition of people not genetically linked [103]. In addition, natural antioxidants (polyphenols, isoflavones, ginsenosides and flavonoids), extracted from plants have a proven antioxidant function and protective effect on mitochondrial function. For example, green tea and polyphenols are believed to be a strong antioxidant against hydroxyl radicals, nitric oxide and lipid oxidation [112,113].

However, due to the limited studies to date, the efficacy and side effects of natural antioxidants for the treatment of CNS diseases have yet to be determined. Molecules, such as vitamins C and E, glutathione, and coenzyme Q10 (CoQ10), lipoic acid and melatonin, play an important role in the endogenous defensive strategy against oxygen FR [114–129]. Mitochondrial gene therapy (studies targeting the amelioration of mtDNA lesion) [130–132], and substitution of defective mtDNA using gene-carrying vectors to send recombinant mtDNA into cells, are other approaches for mitochondrial gene therapy. Novel methodologies for manipulation of mitochondrial drugs are needed for mitochondria-targeted treatment [133–138]. To date, there is little progress in the study of gene therapy for preventing oxidative damage, and therefore clinical trials are necessary for further exploration of this question.

In patients with refractory epilepsy, current treatments include surgical resection of areas affecting epilepsy [139–142] and the ketogenic diet or other alternative diets used since the 1970s [143–146]. However, the refractoriness of epilepsy continues to be an unresolved problem. Some human studies, although still in the experimental phase and without definitive data, suggest that the administration of some antioxidant agents can be used as adjuvants in the treatment of refractory epilepsy. These antioxidant agents include melatonin [147], vitamin E [148], selenium [149,150] and allopurinol [151–156]. It is urgent to shed more light on the mechanisms of refractory epilepsy and find new treatments that could improve the quality of life of patients.

## 5. Conclusions

The evidence from experimental models and patients suggests the participation of oxidative stress in epilepsy, including cases refractory to drug treatment. A broad range of studies has provided evidence of a role of oxidative stress in refractory epilepsy, emphasizing the need for ongoing research in this area, especially the exploration of the physiopathological, biochemical, cellular and molecular mechanisms involved in this drug-resistant pathology. Given the lack of alternatives for patients with refractory epilepsy, the use of antioxidants should certainly be considered as a therapeutic alternative.

## Figures and Tables

**Table 1:** Reactive oxygen species.

Radical	Non-Radical
Superoxide (O_2_^•−^)	Singlet oxygen (^1^O_2_) ^1^Δ and ^1^∑ form
Monoatomic oxygen (O·)	Hydrogen peroxide (H_2_O_2_)
Hydroxyl (HO·)	Ozone (O_3_)
Peroxyl (RO_2_·)	Peroxynitrite anion (ONOO^−^)
Alkoxyl (RO·)	Hypochlorous acid (HOCl)
Hydroperoxyl (HO_2_·)	Hypobromous acid (HOBr)
